# Physiology, phylogeny, early evolution, and GAPDH

**DOI:** 10.1007/s00709-017-1095-y

**Published:** 2017-03-06

**Authors:** William F. Martin, Rüdiger Cerff

**Affiliations:** 1 Institute of Molecular Evolution, University of Düsseldorf, Universitätsstr. 1, 40225 Düsseldorf, Germany; 2 Institute of Genetics, Technical University of Braunschweig, Spielmannstr. 7, 38106 Braunschweig, Germany

**Keywords:** Endosymbiosis, Plastids, Mitochondria, Cell evolution, Peter Sitte

## Abstract

The chloroplast and cytosol of plant cells harbor a number of parallel biochemical reactions germane to the Calvin cycle and glycolysis, respectively. These reactions are catalyzed by nuclear encoded, compartment-specific isoenzymes that differ in their physiochemical properties. The chloroplast cytosol isoenzymes of d-glyceraldehyde-3-phosphate dehydrogenase (GAPDH) harbor evidence of major events in the history of life: the origin of the first genes, the bacterial-archaeal split, the origin of eukaryotes, the evolution of protein compartmentation during eukaryote evolution, the origin of plastids, and the secondary endosymbiosis among the algae with complex plastids. The reaction mechanism of GAPDH entails phosphorolysis of a thioester to yield an energy-rich acyl phosphate bond, a chemistry that points to primitive pathways of energy conservation that existed even before the origin of the first free-living cells. Here, we recount the main insights that chloroplast and cytosolic GAPDH provided into endosymbiosis and physiological evolution.

## Introduction

Peter Sitte was a virtuoso in the art of electron microscopy. He devoted his scientific career to understanding the nature and evolutionary basis of compartmentation in eukaryotic cells and the role that endosymbiosis played therein (Sitte 2007). Thanks mainly to electron microscopic studies in the 1960s and 1970s, scientists in 2016 recognize two kinds of cells: the prokaryotic type and the eukaryotic type. The main difference that distinguishes the two cell types is the nature of internal compartmentation in eukaryotes. The chromosomes in eukaryotic cells are separated from the cytoplasm by membrane surrounding the cell nucleus, while chromosomes in prokaryotes are freely dispersed throughout in the cytoplasm. Eukaryotes typically possess a complex endomembrane system, and mitochondria, plant, and algal cells possess chloroplasts in addition. By the measure of compartmentation, the most complex cells in nature are found among the algae that possess plastids surrounded by three or four membranes, plastids that are remnants of evolutionarily reduced eukaryotic cells residing within the cytosol of another nucleus-bearing cell (Stoebe and Maier 2002; Gould et al. 2008). Though it was not always the case, today, biologists recognize that complexity in eukaryotic cells stems from endosymbiosis (Archibald 2014).

Endosymbiotic theory takes root in Mereschkowsky’s classical essay on the origin of plastids (Mereschkowsky 1905). It has a long and turbulent history, as recently summarized elsewhere (Martin et al. 2015). The elder of us first learned about endosymbiosis in the 1960s in Peter Sitte’s cell biology lectures at the University of Freiburg. Endosymbiotic theory—the prospect that mitochondria and chloroplasts descended from free living prokaryotes that entered into a symbiotic relationship with their respective host cell early in eukaryotic history—was a very exciting, almost revolutionary, prospect in cell evolution that opened up fundamentally new avenues of pursuit to investigate and understand eukaryotic intracellular compartmentation. One aspect in particular was important for endosymbiotic theory: the compartmentation of metabolism in eukaryotes. Early on, endosymbiotic theory had it that the core metabolic functions of mitochondria (respiration) and chloroplasts (photosynthesis) were direct inheritances from the bacterial ancestors of organelles. It was also clear from electron microscopy that organelles possessed DNA (Kowallik and Haberkorn 1971), and that organelle genomes were much too small to encode all of the proteins that underpin respiration and photosynthesis (Herrmann et al. 1975). As a consequence, most of the proteins that support the physiological function of chloroplasts and mitochondria had to be encoded in nuclear chromosomes, which meant that there had to have been some form of gene transfer going on from endosymbionts to the host, or as Wallin put it with regard to mitochondria, “...bacterial organisms may develop an absolute symbiosis with a higher organism and in some way or another impress a new character on the factors of heredity. The simplest and most readily conceivable mechanism by which the alteration takes place would be the addition of new genes to the chromosomes from the bacterial symbiont.” (Wallin 1925; p. 144).

Chloroplast cytosol isoenzymes provided unique opportunities to test crucial predictions of endosymbiotic theory with molecular evolutionary studies. If endosymbiotic theory was correct about chloroplasts arising from cyanobacteria, it followed that the nuclear gene for the chloroplast enzyme should ultimately stem from the genome of the cyanobacterial ancestor of plastids, while the cytosolic enzyme should reflect the evolution of the host cell that acquired the plastid. As it applied to isoenzymes, this specific inference was called the product specificity corollary to endosymbiotic theory (Weeden 1981). Chloroplast cytosol isoenzymes for higher plant glyceraldehyde-3-phosphate dehydrogenases (GAPDH) provided an excellent system to test the prospect of gene transfer in endosymbiosis.

### GAPDH: from protein to DNA

Like endosymbiotic theory, GAPDH itself has a long and turbulent history. The enzymatic activity of GAPDH goes way back, both in plant metabolism and in enzymology itself. Indeed, it was among the first enzymes to be studied. Early work on GAPDH focused on the enzyme from muscle and yeast. In the 1930s, when Embden, Meyerhof, and Parnas were making headway into synthesis of adenylpyrophosphate (now called ATP) from the oxidation of glucose, Dorothy Needham reported that the energy released in the oxidation of triosephosphate indicated “The existence in muscle extract of a coupled mechanism whereby synthesis of adenylpyrophosphate (from adenylic acid and free phosphate) can accompany oxido-reduction” (Needham and Pillai 1937, p. 1850). Otto Warburg reported the crystallization of the yeast enzyme (Warburg and Christian 1939), which he called *das oxydierende Gärungsferment*. By the time that Cori et al. (1948) reported crystallization of the animal enzyme, it was called d-glyceraldehye-3-phosphate dehydrogenase. Stumpf (1950) reported GAPDH activity in plants, noting that the oxidation of fructose-1,6-bisphosphate in pea seedlings occurs by a series of reaction that “is apparently similar to that in yeast and animal tissues.”

A GAPDH activity was reported in chlorophyll-containing plant tissues that required NADPH (Gibbs 1952). Into the 1970s, it was not clear whether one GAPDH enzyme existed in plants that was modified for NADPH-dependent activity (Melandri et al. 1970; Cerff 1974) or whether isoenzymes existed for the NADH- and NADPH-dependent activities and, if the latter case were true, whether such isoenzymes were specific to the GAPDH reactions of glycolysis in the cytosol and the Calvin cycle of plastids. Classical enzymology—separation, purification, and characterization of the plant enzymes (Cerff 1978a, b; Cerff 1982a)—demonstrated the existence of cytosol-specific (NADH dependent) and chloroplast-specific (NADPH accepting) GAPDH isoenzymes in photosynthetic tissues of higher plants (Cerff 1979; Cerff and Chambers 1979). The isoenzymes indeed had very distinct evolutionary histories (Melandri et al. 1970; Cerff 1982a), and they were encoded by nuclear genes (Cerff 1982b; Cerff and Kloppstech 1982).

In the early 1980s, there was a lot of excitement surrounding the newly discovered ability of molecular sequence comparisons and phylogenetic trees, not only to test endosymbiotic theory but to also reconstruct early evolution more generally. Lynn Margulis had very effectively revived endosymbiotic theory (Sagan 1967), but she never made the transition to testing its predictions with molecular phylogenetics. It was Margaret Dayhoff (Schwartz and Dayhoff 1978) who ushered endosymbiotic theory into the era of scientific testing with phylogenetic trees created from protein sequences. Disappointingly, sequences for the plant GAPDH isoenzymes could not be obtained via standard Edman degradation protein sequencing technologies that had worked so well for cytochrome *c* or ferredoxins (Dayhoff 1965). That meant that if one wanted to get the amino acid sequences for plant GAPDH in order to address the decisive evolutionary issues at the forefront of the field, one had to utilize the latest technology: DNA sequencing. At that time, the road to obtaining protein sequences from DNA sequences went through cDNA sequences and antibodies. Methods for separating and purifying the isoenzymes (Cerff 1979) meant that antisera against the purified proteins were available (Cerff and Kloppstech 1982). The availability of antisera permitted use of an archaic, experimentally demanding, but often effective technique called hybrid release translation. If all has gone well, the result of the hybrid translation approach to cloning delivers the desired cDNA clones, and the chemical method of Maxam and Gilbert delivers their sequences (Martin and Cerff 1986).

### The origin of plastids

**Fig. 1:**
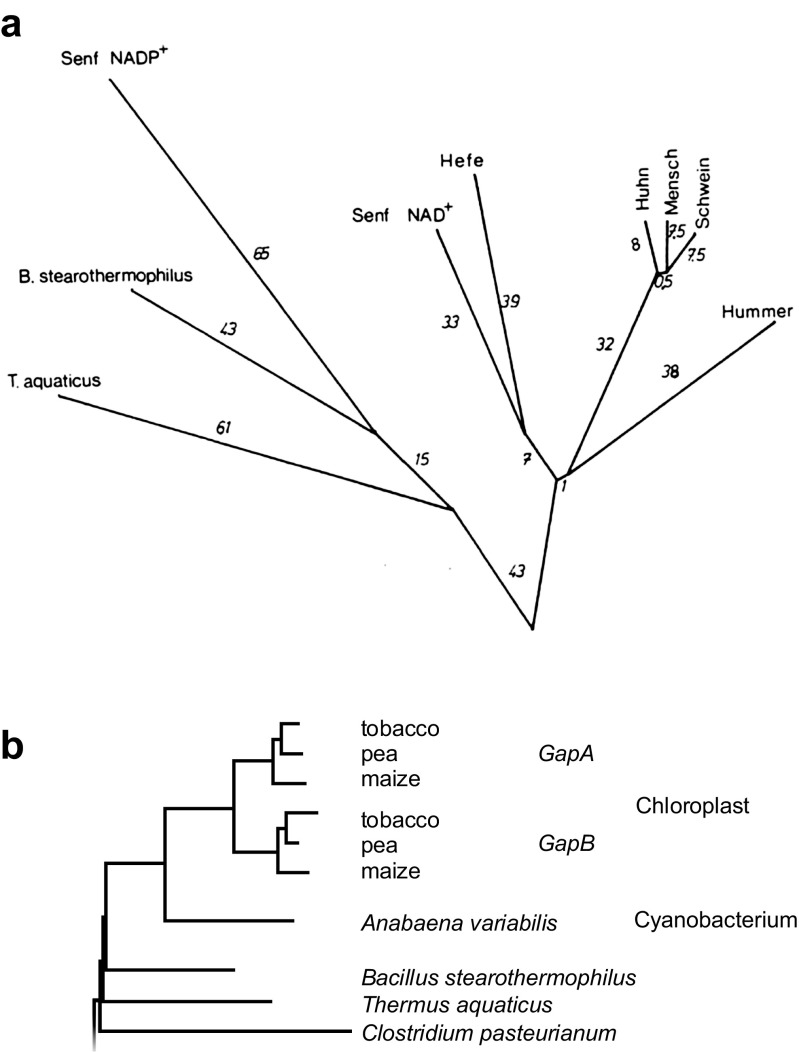
Phylogenetic trees of GAPDH amino acid sequences. **a** The tree was constructed by the method of Fitch and Margoliash (1967) with two interactive rounds of topological adjustment to reduce the sum of squared differences between (i) pairwise distances calculated between amino acids sequences using the minimum mutation distance of Dayhoff (1965) and (ii) pairwise distances measured in the constructed phylogenetic tree (Martin 1985). *Senf*, mustard; *Hefe*, yeast; *Huhn*, chicken; *Mensch*, human; *Schwein*, pig; *Hummer*, lobster; *B*., *Bacillus*, *T*., *Thermus*. *Numbers at branches* are distances. **b** The nuclear encoded genes for the A and B subunits of higher plant chloroplast GAPDH, an A_2_B_2_ tetramer, branch with the Calvin cycle homologue from cyanobacteria. Redrawn from Martin et al. (1993)

With the cDNAs and derived amino acid sequences, we were able to show that the nuclear encoded chloroplast enzyme was more similar to its homologues from bacteria than it was to homologues from eukaryotes, and that the nuclear encoded cytosolic enzyme was more similar to homologues from animals and yeast than it was to homologues from prokaryotes (Fig. 1a). This clearly bore out the predictions from endosymbiotic theory, a novel and exciting find. In the process of not getting our paper published in two journals, however, the sequences of GAPDH from *Escherichia coli* became published, and referees, one intimately familiar with *E. coli* GAPDH, were suddenly demanding that we explain why *E. coli* GAPDH was more similar to eukaryotic sequences than it was to GAPDH from *Thermus aquaticus* or *Bacillus stearothermophilus*. We reasoned that this was also probably a case of lateral gene transfer (our working hypothesis entailed lateral gene transfer anyway), but not from prokaryotes to eukaryotes as in the case of the chloroplast enzyme, rather from eukaryotes to prokaryotes (Martin and Cerff 1986). GAPDH thus presents one of the earliest reports in the literature for sequence-based inference of lateral gene transfer, a concept that a decade later became quite popular (too popular in some circles perhaps), and sequences from other plant sources bore out the predictions of endosymbiotic theory with respect to the plant enzyme (Brinkmann et al. 1987).

Chloroplast GAPDH uncovered additional surprises. The NADPH-utilizing plastid enzyme from higher plants was known to exist in two forms, an A_4_ homotetramer and an A_2_B_2_ heterodimer (Cerff and Chambers 1979). The A and B subunits were shown to be the result of a nuclear gene duplication that took place early in the evolution of the green plant lineage, with the B subunit having acquired a short C-terminal extension with conserved cysteine residues (Brinkmann et al. 1989). The C-terminal extension of GapB was acquired at the beginning of land plant evolution from the nuclear encoded small redox active protein CP12, which was shown to interact with the A_2_B_2_ and A_4_ forms of chloroplast GAPDH in addition to phophoribulokinase in the absence of NADP(H) (Wedel et al. 1997, Wedel and Soll 1998, Petersen et al. 2006a). This interaction blocked CO_2_ fixation activity in the dark and prevented futile cycling between glycolysis and the Calvin cycle. It also explained why the chloroplast enzyme aggregated in the presence of NAD(H), which was the key to efficient separation and purification of the isoenzymes (Cerff 1982a).

### The origin of the first genes

In the early 1980s, before the concept of an RNA world (Gilbert 1986) had been born, people were still vigorously debating the issue of what came first, protein, or DNA. One of the big puzzles was how the first long open reading frames in genes came to be, and how enzyme sized proteins arose in the absence of accurate template replication. Figuring prominently in that debate was Walter Gilbert’s exon theory of genes (Gilbert 1987), according to which introns were relicts from the primordial assembly of genes at life’s origin and that intron positions in modern genes corresponded to the boundaries between structural modules of protein function called domains. Modules, being shorter and easier to evolve, could recombine via “exon shuffling” and exons could perhaps undergo alternative splicing (Gilbert 1978), thereby promoting ancient enzyme diversity. Ancient enzymes such as GAPDH were clearly well suited to test those ideas, and indeed, we found intron positions that were present between the same nucleotides in the same homologous codon in the nuclear gene for chloroplast GAPDH and in animal GAPDH (Quigley et al. 1988). The closer we looked into GAPDH genes, the more evidence we found for identical intron positions in anciently diverged genes (Liaud et al. 1990; Kersanach et al. 1994; Cerff et al. 1994). During those investigations, however, our views concerning the age of eukaryotic GAPDH genes had to be revised, and like so many other things in evolution, cyanobacteria were responsible for the change.

### Endosymbiosis and the unexpected origins of eukaryotic genes

The endosymbiosis story for the origin of chloroplast GAPDH was conceptually satisfying, but an important piece was missing. The prediction from endosymbiotic theory was, namely that the plastid enzyme should be more similar not just to prokaryotic homologues in general, but to cyanobacterial homologues in particular. Therefore, we embarked to obtain the sequence of GAPDH from cyanobacteria, which we did, finding more than we expected. We found that the cyanobacterium that we had investigated, *Anabaena*, indeed had a Calvin cycle homologue of GAPDH that, in phylogenetic trees, branched very specifically with the nuclear encoded chloroplast GAPDH enzymes of higher plants (Fig. 1b), a resounding confirmation of the prediction from endosymbiotic theory (Martin et al. 1993). However, that was not all that *Anabaena* had in store, it also harbored two other GAPDH genes, one of which shed dramatic light on the pesky *E. coli* GAPDH that was annoyingly similar to eukaryotic GAPDH. *Anabaena* had a copy of the same *E. coli* GAPDH gene that, up until then, we had thought to be a eukaryote-to-prokaryote transfer.

**Fig. 2:**
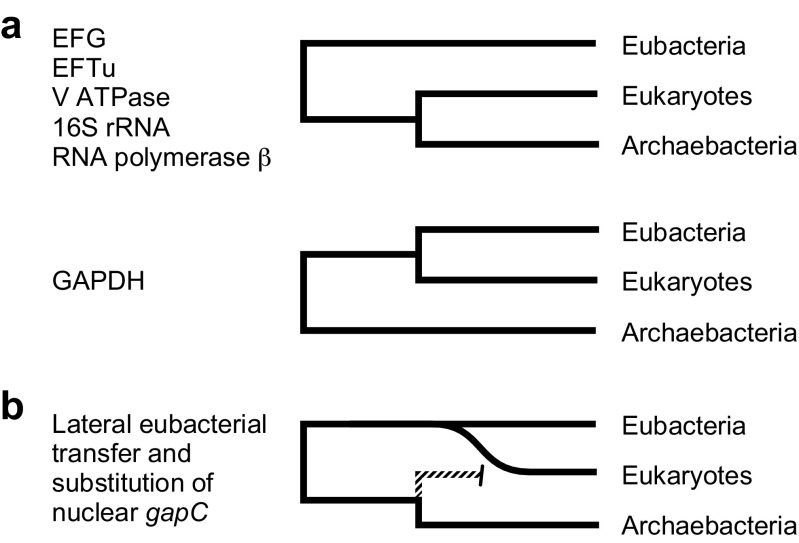
Domain relationships. **a** Schematic representation of domain relationships for several markers used at the time that showed a sister group relationship between eukaryotes and archaea contrasted to the domain relationships depicted by sequences for glycolytic GAPDH sequences. **b** Interpretation of the difference between domain relationships depicted by the data sets. Redrawn after Martin et al. (1993)

We had become pretty good at interpreting trees, but this observation was a puzzle. In order to make sense of it, we had to pay careful attention (i) to what Reinhard Hensel and his team had been finding for archaeal GAPDH (Fabry and Hensel 1988; Zwickl et al. 1990) and (ii) to what everyone was saying at the time with regard to eukaryote origins. In the early 1990s, Woese and Kandler’s rooted three domain tree was as *en vogue* as it gets it biology (Woese et al. 1990). However, it was a tree of ribosomes. The assumption back then, before we had genomes, was that the rRNA tree was speaking for the genome as a whole, and that ultimately, when we had all the data, all genes would tend to paint roughly the same picture as the rRNA tree, with archaea as the sisters to the eukaryotes and with mitochondrion-lacking eukaryotes branching early on the eukaryotic branch, meaning that mitochondria came late in eukaryote evolution and that eukaryotes are, for all practical purposes to be seen as grown-up archaea. Accordingly, eukaryote GAPDH genes should derive from archaeal GAPDH genes. That was not, however, what Reinhard Hensel was finding (Fabry and Hensel 1988; Zwickl et al. 1990). They were finding that archaeal GAPDH was, for all practical purposes, unrelated to eukaryote GAPDH, but it was also just as unrelated to bacterial GAPDH. That indicated a split of archaeal GAPDH from its homologues in the other two domains at the earliest stages of protein evolution, which in itself was not hugely surprising. However, from our standpoint, trying to make sense of data that was fundamentally different from what everyone else was finding (Fig. 2), there was a substantial surprise. If we thought things through in full (which is something we spent a lot of time doing in those days), it also indicated that all of the GAPDH genes that we had seen in eukaryotes up until then were—just like chloroplast GAPDH—acquired from bacteria. Eukaryotic genomes were not generally archaeal, they were chimeric.

Because animals and fungi had never possessed plastids (so says the guiding logic of endosymbiotic theory), this led us to posit that the eukaryotic gene for glycolytic GAPDH is also an acquisition via endosymbiosis and ultimately stems from mitochondrion. Cytosolic GAPDH, a core enzyme of carbon and energy metabolism and the key energy-conserving step of glycolysis, reflected the evolution of the host that acquired the plastid, but not the evolution of the host that acquired the mitochondrion. Endosymbiosis had more to do with compartmentalized energy metabolism in eukaryotes than anyone had suspected. We concluded by saying: “The evidence for eubacterial origin of eukaryotic GAPC, an essential glycolytic enzyme, suggests that eukaryotic genomes are more highly chimeric than previously assumed. Whereas most organellar proteins are currently encoded in the nucleus, endosymbionts may have donated many genes to the nucleus without organellar reimport of the protein, thereby enriching the genetic and metabolic potential of the host” (Martin et al. 1993; p. 8695). We began to notice that endosymbiotic gene transfer and endosymbiotic gene replacement were probably more widespread than anyone had suspected; it was an exciting time.

Not until many years later, with the availability of complete genomes, did people see that the vast majority of eukaryotic genes stem from bacteria, not from archaea (Esser et al. 2004; Pisani et al. 2007; Cotton and McInerney 2010; Ku et al. 2015). Today, we know that eukaryotes are not just grown up archaea, they are the product of endosymbiosis (Zimorski et al. 2014; Martin et al. 2015), but for some reason, it is still very popular to say that eukaryotes arose from archaea or to write about the “archaeal origin of eukaryotes” (Williams et al. 2013; Koonin 2015), even though the statement is, if we look at the data openly, flatly wrong because eukaryotes possess three times more bacterial-derived genes that they possess archaeal derived genes (Esser et al. 2004; Pisani et al. 2007; Cotton and McInerney 2010; Ku et al. 2015). What the data say (Zaremba-Niedzwiedzka et al. 2017) is that the host for the origin of mitochondria has an archaeal origin (McInerney et al. 2014). A democratic genome with one gene one vote would elect the “bacterial origin of eukaryotes.” GAPDH was just the tip of the iceberg.

### The evolution of protein compartmentation during eukaryote evolution

**Fig. 3:**
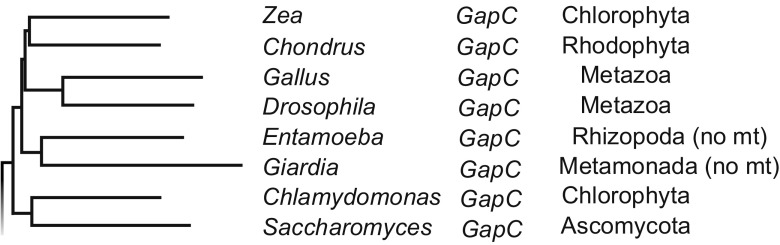
Lineage relationships for GAPDH from eukaryotes lacking typical mitochondria (abbreviated as “no mt”). Redrawn after Henze et al. (1995)

The finding that endosymbionts may have donated many genes to the nucleus without organellar reimport of the protein (Martin et al. 1993) sharpened our awareness to an issue that eventually became quite pressing. We (and everyone else) had been assuming that the products of endosymbiont-derived genes were specific to the organelle from which the gene was acquired; after all, this was what the product specificity corollary to endosymbiotic theory, which we had been testing for a decade said. Cytosolic GAPDH was an exception, and a plastid-targeted version of cytosolic GAPDH was also an exception (Meyer-Gauen et al. 1994; Petersen et al. 2003). When we looked around more generally, almost all of the enzymes of central carbohydrate metabolism in plants were an exception, and it was quickly becoming apparent that eukaryotes had a bacterial glycolytic pathway (Martin 1996), which was also true of eukaryotes such as *Giardia* or *Entamoeba* that lacked typical mitochondria (Henze et al. 1995), as shown in Fig. 3.

All that provided cause to think about the process of endosymbiotic gene transfer as a process in real time. When the bacterial ancestors of chloroplasts and mitochondria became endosymbionts, they did not bring along their own pre-existing protein import machinery. Rather, both the TiM and ToM protein import machinery of mitochondria and the TiC and ToC protein import machinery of plastids are eukaryotic inventions, albeit consisting of some prokaryotic-derived basic components (Soll and Schleiff 2004), arose within the eukaryotic lineage, each following the origin of the respective organelle. The consequence of that insight is that at the onset of mitochondrial endosymbiosis, when the first genes were being transferred to the chromosomes of the host, there was no place for the translated products of endosymbiont-derived genes to go except to the cytosol (or to the host’s plasma membrane). That in turn means that it should not be all that surprising to see a predominance of bacterial genes in eukaryotes. We were assuming 20 years ago that which today’s data say (Zaremba-Niedzwiedzka et al. 2017): The host that acquired the mitochondrion was an archaeon, a prokaryote. Gene transfers during endosymbiosis transformed the archaeal host from within because if the first transfer did not work (that is, if it just gave rise to a pseudogene), then maybe the next transfer would be successful, or maybe the next after that.

At the origin of eukaryotes, the mitochondrial endosymbiont’s genome was a continuous and persistent source of new genes for the host. At the onset of that symbiosis, however, the products of the first transferred genes became targeted to the host’s cytosol. Only after the symbiotic consortium had evolved the TiM and ToM complexes in the two mitochondrial membranes could the products of transferred genes be reimported. The evolution of the TiM and ToM complexes fostered the evolution of N-terminal transit peptides that would direct gene products faithfully to mitochondria. GAPDH shed light on that process, too, and introns were once again involved. Walter Gilbert’s team found a case in which the NAD-binding domain of cytosolic GAPDH had been appended to the N-terminus of plant cytochrome *c* via exon shuffling to generate a functional transit peptide (Long et al. 1996). Thus, while chloroplast GAPDH possessed transit peptides, cytosolic GAPDH in plants provided genetic starting material for the generation of transit peptides, yet not de novo, rather by exon shuffling.

That was all extremely helpful for understanding the situation, but some old important questions remained and new ones arose. Among the old important questions was the issue of why, with all that transfer going on, any genes remained in organelles at all, which John Allen’s corr hypothesis explains better than any other competing theory, it has to do with the need to maintain redox balance in bioenergetic membranes, which requires that components of the electron transport chain be encoded in the organelle (Allen 2015).

Among the new questions to unfold from those insights was the issue of why eukaryotes, which everyone thought descend from archaea (say ribosomes), should have a bacterial glycolytic pathway in the first place instead of their original archaeal glycolytic pathway. Keeping the bigger picture in focus—and remaining critical of the theoretical foundations upon which the whole endosymbiosis endeavor was resting—perhaps the answer was that something else even more fundamental was wrong with endosymbiotic theory. What was wrong? As long as glycolysis had been known and for as long as anyone ever thought that mitochondria were endosymbionts, everyone had always assumed that the host for the origin of the mitochondrion was a heterotroph. What if the host was an autotroph? An autotroph would actually require a new glycolytic pathway in order to supply its mitochondria with carbon substrates because in autotrophs, carbon flux is specialized and optimized to run from pyruvate to glucose. In order to feed a mitochondrion in the cytosol of an autotroph, a typical bacterial Embden-Meyerhof catabolic carbon flux was needed as it occurs in all eukaryotes. Stumpf's (1950) inference about glycolysis in plants being “similar to that in yeast and animal tissues” applied to all eukaryotes in general. Glycolysis was present in the eukaryotic common ancestor, but was it present in the host? At the same time, there were the anaerobic forms of mitochondria out there for which endosymbiotic theory also did not account, hydrogenosomes (Lindmark and Müller 1973; Müller 1988).

If the host was an autotrophic archaeon, then it would be dependent upon H_2_, like most archaeal autotrophs are (Fuchs 2011). If it were dependent upon H_2_ produced by the common ancestor of mitochondria and hydrogenosomes, that would provide a selective pressure to associate the endosymbiont to its host (Martin and Müller 1998). Finally, we had a version of endosymbiotic theory where the host actually needed its endosymbiont, but a very different version than had ever been out there, one in which by gene transfer, the symbiont’s bacterial glycolytic pathway ended up in the cytosol, feeding the mitochondrion, and converting the autotrophic host cell compartment into a heterotrophic cell compartment (Martin and Müller 1998). Not only would a bacterial glycolytic pathway in eukaryotes make sense, but so would the widespread distribution of anaerobic pathways in mitochondria (Müller et al. 2012). In terms of physiology, and in terms of visible syntrophic microbial interactions (Fenchel and Finlay 1995), an autotrophic host for the origin of mitochondria works much better than the phagocytosing host that everyone had always assumed.

While microbiologists tended to like the idea (Madigan et al. 2012), in evolutionary circles, the price for a physiologically based understanding of the prokaryote-to-eukaryote transition was steep. It meant that (i) all of Margulis’s versions of endosymbiotic theory (Margulis et al. 2006) were wrong, that de Duve’s interpretations of hydrogenosomes were wrong (de Duve 2007), that Woese’s three domain tree (Woese et al. 1990) was wrong, and that Doolittle's (1998) view of unrestricted and *continuous* interdomain lateral gene transfer was also wrong. That was a lot of authority being wrong all at once. However, they did not agree on anything among themselves either, and our intent was to get the science right, not to prove particular scientists right. Looking at the matter openly, everyone was trying to account for their own favorite explananda (flagella, peroxisomes, one branch in one tree, and many branches in many trees). We were trying to account for physiology, the way that cells stay alive in their environment, on the simple but robust premise that chloroplasts and mitochondria were once free-living bacteria. The object of our undertaking was to obtain some insights into cell evolution from the starting point of an important enzyme central to carbon and energy metabolism. Nature does not divulge secrets about early evolution lightly, regardless of whether one sets the lever on physiology, or elsewhere.

### Secondary endosymbiosis among the algae with complex plastids

We learned from GAPDH inter alia that when it comes to endosymbiotic gene transfer, there is no homing device that directs the product of transferred genes back to the organelle from which the gene stems, rather it is up to natural variation and natural selection to determine where the gene product will end up. There was once a debate about protein targeting initiated by de Duve, who argued that peroxisomes were once endosymbionts like mitochondria, because how else (so his argument) could one get an entire pathway into a new compartment? Following de Duve’s reasoning for the symbiotic origin of peroxisomes (de Duve 1969), Michels and Opperdoes (1991) explained why specific targeting of an entire pathway to a new organelle does indeed seem very unlikely at first sight, it is because of this: Gene duplication and mutation can readily lead to the signal needed to direct one enzyme to a new compartment, but by itself, the new enzyme is useless, for lack of substrate, for lack of downstream product conversion, or both; hence, the new targeting variant will be a burden for the cell and will be lost. However, if we consider the possibility that protein targeting within eukaryotic cells might not always be 100% specific, with minor amounts of many proteins constantly being imported into the wrong organelle, then even the retargeting of entire pathways during eukaryote evolution does not seem so difficult (Martin 2010) because a very small amount of enzyme can go a long way in terms of activity, and a very small amount of an entire pathway provides a unit of function upon which selection can act. Nowhere in nature is the issue of cell compartmentation more complex than in algae.

The concept of secondary endosymbiosis goes back to Robert E. Lee (1977) and to Sarah Gibbs (1978). It posits that—to use Sitte’s term—the *complex* plastids of algae descend from the engulfment of a eukaryotic alga by a eukaryotic host; such plastids are surrounded by three or four membranes. Genes like that for chloroplast GAPDH that stem from the cyanobacterial ancestor of plastids should also reflect plastid phylogeny, but having been transferred twice during evolution: once during primary endosymbiosis from the cyanobacterial ancestor of plastids to the nucleus of the host and a second time from the nucleus of the algal endosymbiont to the nucleus of its host. GAPDH genes from red algae (Liaud et al. 1994) were extremely insightful in this respect as they traced the red algal ancestry of plastids across the breadth of the photosynthetic eukaryotes having red complex plastids (Liaud et al. 1997; Liaud et al. 2000). GAPDH genes helped discriminate between competing hypothesis for the highly debated number of secondary symbioses involving red algal endosymbionts that occurred during eukaryotic evolution (Petersen et al. 2006b), and they helped to clarify the evolutionary origin of land plants themselves (Petersen et al. 2006a). Rounding out the family of plant GAPDH, there was also a non-phosphorylating form of the enzyme, called GAPN, that turned out to be a member of the aldehyde dehydrogenase family, which did have homologues among archaea, providing additional insights into the early diversification of genes in the last universal common ancestor of all life (Habenicht et al. 1997).

The reason for bringing GAPDH out of the cold room and into the realm of molecular biology in the first place was to discriminate between competing hypotheses. Among the algae with complex plastids, the numbers of possible endosymbiotic events and gene transfers that one can infer from GAPDH gene phylogenies can become bewildering. In trying to reconcile the observed phyletic patterns with the biology of the organisms—the explanandum of the intellectual exercise—it is easy to lose sight of the forest (the biology) for the gene trees. Gene trees are helpful when it comes to hypothesis testing, and they neither have the same weight, as observations in nature, nor are they even in the same category. The algae possessing GAPDH genes and the GAPDH genes themselves are real in the sense that they exist in nature. By contrast, branches in gene trees are not real. Branches in trees come from computer programs (or in the case of Fig. 1, from a pencil, pen, slide rule, and Letraset). In order to bring the branching patterns in gene trees into logical agreement with what we think true species relationships should be, one only needs to add four ingredients: phylogenetic error, gene losses, endosymbioses, and (since our 1986 paper anyway) lateral gene transfer.

In the absence of a priori knowledge about the subject of inquiry, for whose study the tree was constructed, there is no simple way to decide how much of which ingredient to add. That calls for a sound philosophical foundation for the scientific enquiry at hand, something in which the elder of us amply received formal training during a decade of research in Freiburg. The philosophy of science as it applies to practical empirical inquiry is something about which we often discussed in Hannover and Braunschweig, especially when it was time to think things through in full, in the hope of extracting *belastbare* [Ger: sturdy in the sense of being able to support something] insights into early evolution from protein sequence data. Without going into justification, detail or digression, two sets of guidelines always seemed helpful.

One is the principle of Occam’s razor as splendidly defined in Webster’s 9th Collegiate Dictionary: “a scientific and philosophic rule that entities should not be multiplied unnecessarily which is interpreted as requiring that the simplest of competing theories be preferred to the more complex or that explanations of unknown phenomena be sought first in terms of known quantities*.*” The last 12 words are the most important. They set Occam’s razor miles apart from mere parsimony, and in practical terms, they translate to a useful doctrine for inference: Do not make up anything unless it is absolutely necessary and work with observations in nature.

Another set of reasoning guidelines can be found in a little noted paper by Lee about the number and nature of plastid symbioses: “Any evolutionary scheme should adhere to the following three principles. (1) A monophyletic origin of any organism, chemical compound or cytoplasmic structure has the greatest statistical probability of being correct. (2) The loss of a non-essential structure can require just the mutation of a single gene but the acquisition of a structure generally requires many mutations and a considerable amount of time. (3) Most organisms in evolutionary sequences would have been lost, yet in postulating phylogenetic events the plausibility of the theory can be enhanced by the existence of organisms similar to those in the proposed scheme*.*” (Lee 1972, p. 44). Many of today’s papers about lateral gene transfer among eukaryotes could benefit from Lee’s rules, no. 2 in particular.

### Thioesters, energy, and chemical relicts from life’s origin

**Fig. 4:**
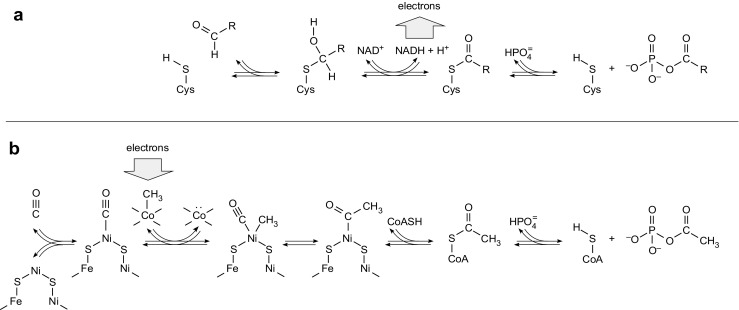
Energy conservation as high-energy phosphate bonds. **a** Mechanism of the GAPDH reaction in the oxidative direction, redrawn after Segal and Boyer (1953) and Biesecker et al. (1977), R = CHOHCH_2_OPO_3_^2−^. The *vertical arrow* underscores the oxidative nature of the reversible reaction when drawn from left to right. For sustained flux in that direction to allow sustained synthesis of high-energy phosphate bonds, suitable electron acceptors must be available in the environment. These acceptors could not have been generated fermentatively during metabolism of organics from space because organics from space are unfermentable substrates (Schönheit et al. 2016). Assuming the (debateable) existence of a primordial source of sugars, oxidative energy conservation requires the existence of oxidants as electron acceptors; oxidants are however thermodynamically unfavorable for the accumulation of the building blocks of life (see text). Under physiological conditions, the acyl phosphate bond in the rightmost compound, 1,3-bisphosphoglycerate, has a free energy of hydrolysis of −52 kJ/mol (Thauer et al. 1977). **b** Reductive synthesis of acyl phosphate (acetyl phosphate) from CO and a methyl group as it occurs in the acetyl-CoA pathway of acetogens in (Fuchs 2011; Schuchmann and Müller 2014) and in some methanogens when grown on CO (Rother and Metcalf 2004). The reactions are drawn from data compiled in Svetlitchnaia et al. (2006) AND in Ragsdale (2009) particularly Figs. S5 and S6, in Fuchs (2011), and in Schuchmann and Müller (2014). The *vertical arrow* indicates that the exergonic nature of the reaction sequence, hence its ability for sustained synthesis of acyl phosphate, entails the continuous reduction of CO_2_ and hence requires the environmental availability of a suitable reductant such as H_2_, which was abundant on the early Earth (Sleep et al., 2011) and is still abundant today in hydrothermal vents (McCollom and Seewald 2013). Electrons from the H_2_/H^+^ couple, which has a standard midpoint potential of −414 kJ/mol at pH 7 (Thauer et al. 1977), are used by hydrogenotrophic acetogens and methanogens to synthesize CO and the methyl group from CO_2_ (Fuchs 2011, Schuchmann and Müller 2014). Under physiological conditions, the acyl phosphate bond in the acetyl phosphate has a free energy of hydrolysis of −45 kJ/mol (Thauer et al. 1977). In microbial in metabolism, both 1,3-bisphosphoglycerate and acetyl phosphate typically phosphorylate ADP to ATP, which has free energy of hydrolysis of −32 kJ/mol (Thauer et al. 1977), via substrate level phosphorylation. Some readers might object to the use of the terms energy-rich bond or high-energy bond, but the terms are very useful and the lability of the bonds in question is founded in the circumstance that the valence electrons of P and S are in the third shell, which can accept further electrons in the *d* orbital, and in the circumstance that corresponding bonds have substantial bond lengths, offering ample opportunity for attack by molecules such as water that possess lone electron pairs (Wald 1964)

GAPDH catalyzes a well-studied chemical reaction; the mechanism of which is of interest (Segal and Boyer 1953, Biesecker et al. 1977). In the oxidative direction, the aldehyde carbon in d-glyceraldehyde-3-phosphate is attacked by the thiol of Cys^149^ (*Bacillus stearothermophilus* residue numbering) forming a covalently enzyme-bound hemithioacetal. Hydride removal by NAD^+^ oxidizes the substrate’s thiol-bonded carbon atom to generate a thioester, which harbors an energy-rich bond (Lipmann 1941) with a free energy of hydrolysis of −32 kJ mol^−1^ (Buckel and Eggerer 1965). The substrate is however not removed from the enzyme by hydrolysis, which would release the energy as heat, rather phosphorolysis releases 1,3-bisphospho-d-glycerate. The acyl phosphate bond of 1,3-bisphospho-d-glycerate conserves the energy; it has a free energy of hydrolysis on the order of −52 kJ mol^−1^ (Meyerhof 1951, Thauer et al. 1977) sufficient to readily phosphorylate ADP to ATP (free energy of hydrolysis −31 kJ·mol^−1^, Thauer et al. 1977) via substrate level phosphorylation (Fig. 4a).

Precisely this sequence of reactions from thioester to acyl phosphate (a mixed anhydride) to the β-γ phosphoanhydride bond in ATP has long been thought to be a relic from the very beginning of metabolism. De Duve (1991) suggested that such a thioester–acyl phosphate–ATP conversion, as it occurs in glycolysis, might represent the primordial pathway of energy conservation, but he was looking at the problem of early evolution from the standpoint of sugar oxidations. The problem with his proposal is not the basic chemistry. Rather, the problem is the surrounding environment supporting such a reaction sequence. Like generations of scientists before him (Haldane 1929; Wald 1964), de Duve was assuming that life started off with fermentations. That seems like a harmless assumption, but if we think it through in full on the basis of what we know today about microbial physiology, it turns out to be altogether untenable (Schönheit et al. 2016). For primordial energy conservation in the oxidative direction of the GAPDH reaction, the main problems are twofold. First, on the early Earth, there is no evidence to suggest the existence of any spontaneously synthesized reservoir of any particular sugar isomer, nor is there reason to assume that such a mountain of sugar even existed. This is the same problem encountered when trying to harness energy in some manner from organics delivered to Earth from space: The organic compounds in carbonaceous chondrites are not only extremely heterogeneous in structure (Sephton 2002) so that no specific isomer could become the focus of biological energy conservation, but they are also unfermentable substrates (Schönheit et al. 2016), meaning that the only way to harness energy from them is through oxidation, which requires an oxidant, an electron acceptor like O_2_ or Fe^3+^ or similar.

The need for an oxidant brings us to the second problem. The invocation of oxidants, such as Fe^3+^ as the original electron acceptors for the oxidative synthesis of thioesters via a reaction similar to that catalyzed by GAPDH (de Duve 1991), has an often overlooked negative consequence of general significance. In the presence of strong oxidants like Fe^3+^, carbon equilibrium lies on the side of CO_2_ rather than on the side of reduced carbon compounds, the building blocks of life (Sousa et al. 2013, Amend and McCollom 2009). Fe^3+^ is a strong oxidant. The midpoint potential, *E*_*0*_’, of the Fe^3+^/Fe^2+^ couple is +772 mV, that of the O_2_/H_2_O couple is +818 mV (Thauer et al. 1977). At the onset of biochemical energy harnessing (or at the origin of life, as one prefers), the first and foremost hurdle is the generation and accumulation of reduced organic compounds comprising the substance of cells, not their conversion to CO_2_. Neither disproportionations (fermentations) nor oxidation of reduced organic compounds offer a tenable path to get started at biochemical origins.

When navigating the waters of early evolution, physiology is a good compass. Seen as a chemical process, the energy metabolic component of physiology entails focusing the flow of a (modestly) specific spectrum of substrates though increasingly narrow channels towards very specific exergonic reactions where energy is conserved. On the early Earth, the main form of carbon was CO_2_ (Sleep et al. 2011). We can say that because the early Earth went through a phase where the planet was molten rock, which is hotter than 1000 °C. Carbon in contact with a global ocean of magma exists as CO_2_, not as glucose or anything similar. When the Earth cooled, CO_2_ remained as the main carbon species. That is good in a physiological sense because it provides a very specific substrate for core physiological reactions.

The surprising aspect for many observers is that under strictly anaerobic conditions, which existed on the early Earth, biological systems can actually harness energy from CO_2_, without light, when using H_2_ as the electron source: Acetogens (Schuchmann and Müller 2014) and, under some conditions, methanogens such as *Methanosarcina acetovorans* growing on CO (Rother and Metcalf 2004) couple the exergonic reduction of CO_2_ with H_2_ to the synthesis of ATP. That exergonic process of ATP synthesis entails the same sequence of substrate level phosphorylation involving thioester–acyl phosphate–ATP conversion as de Duve had in mind. However, neither the substrate level phosphorylations that occurs in acetogens and methanogens nor the enzymes involved have anything to do with glycolysis (Fig. 4b). Energy is released in the reductive direction because in the reaction of H_2_ with CO_2_, the chemical equilibrium lies on the side of reduced carbon compounds (Shock and Boyd 2015). The H_2_ required to drive such reactions in the direction of CO_2_ reduction was present on the early Earth, in abundance, supplied through a geochemical process called serpentinization, which has been generating H_2_ in hydrothermal systems in millimoles per liter amounts since there was water on Earth (Sleep et al. 2004), and still does so today (McCollom and Seewald 2013). Acyl phosphates might have even been the ancestral energy currency of life, more ancient than ATP (Martin 2012). Indeed, their synthesis from methyl groups and CO in anaerobic autotrophs entails reactions catalyzed by transition metals only (Sousa et al. 2013), pointing to a very ancient role for transition metals in biochemical evolution, something that biologists have always suspected (Eck and Dayhoff 1966). In support of that view, reconstructions of early physiological evolution from investigation of ancient genes (Weiss et al. 2016) provide genome-based evidence that the common ancestor of all life conserved energy from the transition metal-dependent reduction of CO_2_ with electrons from H_2_.

Thus, from the perspective of thermodynamics, natural H_2_-producing geochemical processes could have enable reactions involving the synthesis of small molecular weight carbon compounds from CO_2_ to be coupled to the exergonic synthesis of high-energy phosphate bonds. A reaction sequence of that type could have resided at the origin of physiological energy conservation. If so, thioesters would have preceded acyl phosphates in evolution and acyl phosphates would have preceded ATP as the universal energy currency. That would make sense because it means that the simplest form of ATP synthesis, substrate level phosphorylation, would have preceded chemiosmotic ATP synthesis, which requires proteins. From that would follow that the early history of biological energy conservation is recapitulated during every single catalytic cycle of the GAPDH reaction in the oxidative direction, whereby in early energy conservation the reductive synthesis of thioesters from H_2_ and CO_2_ and their conversion to acyl phosphates would have been exergonic, with a coupling of energy metabolism to autotrophy.

### Envoi

Early evolution will always hold a deep element of fascination. After all, it is almost impossible to imagine how anything as complicated as life could get started from anything as simple as rocks, water, and CO_2_ on the early Earth. People look at early evolution from many different angles, from chemical traces for early life to replication, to genetics, to self-organization, and finally to the origins of free-living prokaryotes that almost 2 billion years ago gave rise to eukaryotes—our ancestors—and true biological complexity via endosymbiosis. What many observers fail to note is that life is a chemical reaction, an energy-releasing chemical reaction. The substance of life, and we ourselves, are ultimately just side products of that reaction, whose meaning is to keep the reaction going and whose prerogative is to ponder the origin of it all.
